# Synthesis of plate-like β-tricalcium phosphate nanoparticles and their efficiency in remineralization of incipient enamel caries

**DOI:** 10.1007/s40204-019-00126-y

**Published:** 2019-12-07

**Authors:** Roghayyeh Marefat Seyedlar, Mohammadbagher Rezvani, Samira Barari, Mohammad Imani, Azizollah Nodehi, Mohammad Atai

**Affiliations:** 1 Iran Polymer and Petrochemical Institute (IPPI), Pajouhesh Blvd., Karaj Highway, P.O. Box 14965-115, Tehran, 1497713115 Iran; 2 Surgery Department, Shahed University, Faculty of Dentistry, Tehran, Iran

**Keywords:** Nanoparticle, β-Tricalcium phosphate, Platelet morphology, Incipient enamel caries lesions, Remineralization

## Abstract

The purpose of this study was to synthesize nano-sized β-tricalcium phosphate (nano-TCP) particles and determine its concentration-dependent properties on incipient enamel caries lesions. Nano-TCP was synthesized as a wet chemical through a method using low concentration of precursors and low addition rate of calcium nitrate tetrahydrate as a second phase. Morphology and phase composition of the particles were analyzed by SEM, XRD, and EDXA techniques. Incipient enamel lesions were created in human premolars with an acidic buffer. The teeth were then incubated in aqueous dispersions of nano-TCP as remineralization solutions. Sodium fluoride solution and deionized water were used as positive and negative control groups, respectively. The quality and thickness of the remineralized layer on enamel were investigated using SEM. The data were statistically analyzed by analysis of variance (ANOVA) and post hoc Tukey’s test. The synthesized nano-TCP mostly consisted of porous platelet-like crystals of 50–100 nm thickness and pore diameters of 100–300 nm. SEM observation showed that a homogenous layer was formed on the surface of the enamels remineralized in nano-TCP solutions. The thickness of the mineralized layer was dependent on the incubation time and nano-TCP concentration.

## Introduction

With deeper knowledge gained on processes involved in formation of dental caries and the factors affecting the development of caries lesions in tooth structure, it has been revealed that the lesions are reversible in early stages through applying appropriate changes to the dynamic equilibrium of demineralization/remineralization process (Peters 2010; Selwitz et al. 2007; Young and Featherstone 2010). In the presence of fluoride, the enamel crystals that are mainly composed of hydroxyapatite [Ca_10_(PO_4_)_6_(OH)_2_] are replaced by fluorapatite or fluorohydroxy apatite, which makes enamel more resistant against caries. In addition, fluoride facilitates re-precipitation of the released calcium and phosphate ions on the remaining crystals and prevents leaching them from the tooth (González-Cabezas 2010; Selwitz et al. 2007; Peters 2010; Young and Featherstone 2010). However, the side effects such as acute toxicity and fluorosis can occur after ingesting high doses of fluoride over time periods (Kanduti et al. 2016). Therefore, legal limitations have been developed for high concentrations of fluoride used in dental hygienic products (Roberts 1995). Hence, the fluoride-based hygienic products and also other calcium phosphate-based remineralizing products have been the focus of a wide range of investigations during the recent years (Cochrane et al. 2010; Karlinsey et al. 2010).

Among many materials explored to provide bioavailable calcium and phosphate ions for the remineralization process, crystalline calcium phosphates such as nano-hydroxyapatite (Ebadifar et al. 2017; Mielczarek and Michalik 2014), β-tricalcium phosphate (β-TCP) (Karlinsey and Pfarrer 2012), amorphous calcium phosphate (ACP) (Mundorff-Shrestha et al. 1998; Thompson et al. 1999), calcium sodium phosphosilicate bioactive glass (Novamin) (Burwell et al. 2009), and casein phosphopeptide amorphous calcium phosphate (CPP-ACP) (Cochrane et al. 2012; Li et al. 2014) have drawn considerable attention due to their biocompatibility, chemical stability, wear resistance, and chemical and structural similarity to natural enamel hydroxyapatite (Cochrane et al. 2010). The main limitation of the calcium salts in oral environment is, however, their low solubility, which limits their bioavailability (Eden 2016). More recently, calcium phosphate nanoparticles have been utilized in remineralization of dental defects (Huang et al. 2009; Karlinsey and Mackey 2009). High stability in body temperature, pH (above 4.3), and physiological liquids are the problems associated with the nano-hydroxyapatite (Cochrane et al. 2010), while the presence of bioavailable calcium and phosphate ions in the reduced pH (< 5.5) is necessary for remineralization of enamel caries lesions.

Seeking for calcium phosphates with higher solubility profile in the critical pH of incipient enamel caries lesions, nano-sized β-tricalcium phosphate (nano-TCP) with a higher dissolution rate than hydroxyapatite (Link 2008; Marefat Seyedlar et al. 2014) was synthesized and examined here. Nano-TCP particles with their higher surface area and platelet-like morphology were postulated as good candidates to provide higher dissolution rates in addition to their significant biological activity (Kalita et al. 2007). Their nano sizes may also provide better penetration into the subsurface caries lesion.

Conventionally, β-TCPs are synthesized either through solid-state or wet-chemical methods. The present work focuses on the synthesis of nano-TCP particles by a wet-chemical method, i.e., controlled chemical precipitation through mixing of aqueous solutions of diammonium hydrogen phosphate and calcium nitrate precursors. It is possible to control the nano-TCP particles size, size distribution, morphology, and agglomeration simply by adjusting precursor’s concentration, second phase addition rate, the reaction temperature, pH, and calcination temperature (Kalita et al. 2007). The effects of the reaction parameters such as reaction and calcination time on the nano-TCP morphology and particle size were also studied. The formation mechanism of the particles was investigated by XRD, SEM, and EDXA. Solutions containing the nano-TCPs were then prepared to investigate their effects on the remineralization of incipient enamel caries lesions of human premolar teeth as a function of concentration and application time by measuring the thickness and depth of the lesion with SEM and calcium and phosphate ion concentrations with EDXA.

## Materials and methods

### Materials

Diammonium hydrogen phosphate (DAHP, (NH_4_)_2_HPO_4_), calcium nitrate tetrahydrate (CN, Ca(NO_3_)_2_·4H_2_O), calcium phosphate, calcium chloride (CaCl_2_), potassium dihydrogen phosphate (KH_2_PO_4_), sodium fluoride (NaF), lactic acid, and ammonium hydroxide (NH_4_OH) solution were purchased from Merck (Germany).

### Synthesis of nano-TCP

DAHP and CN solutions were separately prepared by dissolving the salts in distilled water at room temperature as tabulated in Table 1. The CN solution was added dropwise to DAHP solution at a rate of 0.7 mL/min at 33 °C. The solution pH was adjusted by adding ammonium hydroxide and stirred by mechanical stirring for 24 h. The precipitate was then separated from the mixture by filtration through a filter paper and then dried at 80 °C for 24 h in an oven (UNITEMP, LTE, Germany). The dried precipitate was then calcined with heating and cooling rates of 5 °C/min.

**Table 1:** Sample characteristics of nano-TCP powders synthesized at different conditions

Sample code	DAHP conc. (M)	CN conc. (M)	pH	Ripening time (h)	Calcination		Ca/P
					Temp. (°C)	Time (h)	
Nano-TCP-1	0.05	0.075	7.3	24	800	5	1.33
Nano-TCP-2	0.05	0.075	7.3	24	900	2	1.3
Nano-TCP-3	0.05	0.075	8	24	800	5	1.51
Nano-TCP-4	0.05	0.075	7.5	48	800	5	1.457

### Preparation of demineralization and remineralization solutions

An acidic solution with a composition similar to human saliva and reduced pH was prepared to mimic the initiation stage of enamel demineralization and to induce incipient enamel caries lesions; composing 8.7 mmol/L CaCl_2_, 8.7 mmol/L KH_2_PO_4_, 75 mmol/L lactic acid, and 0.05 ppm NaF. The pH was adjusted at 4 using a 5 wt% KOH aqueous solution. For daily pH cycling, a demineralization solution composed of 3.0 mmol/L CaCl_2_, 1.8 mmol/L KH_2_PO_4_, 0.1 mmol/L lactic acid, and 1 wt% carboxymethyl cellulose was prepared and its pH was adjusted at 4 using a 5 wt% KOH aqueous solution (Zero 1995). The remineralization solution was also prepared as 1.2 mmol/L CaCl_2_, 0.72 mmol/L KH_2_PO_4_, 2.6 μmol/L sodium fluoride, 50 mmol/L HEPES ((4-(2-hydroxyethyl)-1-piperazine ethanesulfonic acid)) buffer and its pH was adjusted at 7 using the KOH solution (Langhorst et al. 2009). Methyl paraben (sodium salt) (0.02 g/L) was added to the remineralization and demineralization solutions as a preservative agent.

### Characterizations

X-ray diffraction (XRD: 1500 V, FK 60-04, Germany) pattern of the synthesized nano-TCP samples was obtained from 2*θ* = 5°–70° at ambient conditions using a Siemens D5000 powder XRD system equipped with a FK 60-04 air insulated XRD tube with CuK*α* radiation anode. Morphology and particle size of nano-TCP particles were determined by scanning electron microscopy (SEM, VEGA II, TESCAN, Czech Republic). Ca/P ratio was determined by elemental analysis using energy dispersive X-ray analysis (EDXA: INCA, Oxford Instrument, UK).

### Remineralization studies

The human premolars were cleaned from all soft tissues and debris and observed under stereo microscope (Carton Optical Industries, Thailand) with magnification 2 × to select teeth without any crack, caries, wear, hypoplasia, and decalcification areas. The teeth were kept in 0.1% aqueous thymol solution at 4 °C. Tooth crowns were then cut using a cutting disk (W&H, Austria) under running water. Buccal halves were fully covered with nail polish to block the liquid absorption except a 3 mm × 3 mm window left uncovered on the middle part of the labial surface. The window was polished with fine grain pumice powder and prophylactic brush in a slow-speed hand piece (W&H Austria) for 5 s.

All the specimens (*n *= 63), except 3 as an index of natural tooth, were separately incubated in 40 mL of demineralization solution for 72 h using an incubator (S.57, Shimi Fann, Iran) operating at 37 °C and 100% relative humidity to induce incipient enamel caries lesions. After this step, three teeth specimens were excluded as control group of caries induction step. The remaining teeth specimens (*n *= 60) were randomly divided into five groups that were then treated as follows:

- Group I: positive control (0.05 wt% NaF solution) (*n *= 12)
- Group II: negative control (deionized water (0 wt% nano-TCP solution)) (*n *= 12)
- Group III: 1 wt% nano-TCP solution (*n *= 12)
- Group IV: 3 wt% nano-TCP solution (*n *= 12)
- Group V: 5 wt% nano-TCP solution (*n *= 12).

The pH of all groups was adjusted at 7 by adding NH_4_OH solution.

### pH cycling

The experimental groups were treated in a daily pH cycling regime. To this end, specimens were separately immersed in 10 mL of test solutions (groups I–V) for 15 min so that the windows created on the teeth surface were covered by the solution. After withdrawing of the specimens, they were washed by immersing in distilled water for 15 min to remove excess of the solution. Surface water was then blotted using a fiber-free filter paper just next to the open window. Then, specimens were immersed separately in 20 mL of demineralization solution (2 mL/mm^2^ of enamel surface) for 3 h (Langhorst et al. 2009). The specimens were washed and dried again according to the aforementioned procedure and immersed in 20 mL of remineralization solution for the remaining time up to 24 h. These pH cycles continued for 16 days with daily solution replacement. At the end of the days 4, 8 and 16, three specimens of all test groups were picked up randomly for the subsequent tests. The teeth were sectioned into two buccal halves using paper disk in slow-speed hand piece (Austria, W&H) under running tap water and breaking by a wedging force of a spatula from the back part of the window to avoid the cross section being ground by rotary device.

### SEM analysis

After finishing pH cycling, specimens were dried in an incubator at 37 °C (Incucell, MMM Med center Einrichtungen GmbH, Germany) for 14 days. Then, the morphology of the surface and the thickness of the formed surface layer and caries depth in the cross-sections of specimens were determined by SEM at the magnifications of 5000 and 1000, respectively.

The concentration ratio of calcium and phosphorus in the surface layer, carious lesion body, and underlying intact enamel was detected in the specimens of the day 16 by EDXA.

### Statistical analysis

Statistical analyses were performed using analysis of variance (ANOVA) and Tukey’s post hoc test. Differences were considered statistically significant when the *p* value was < 0.05.

## Results and discussion

### Nano-TCP synthesis

**Fig. 1:**
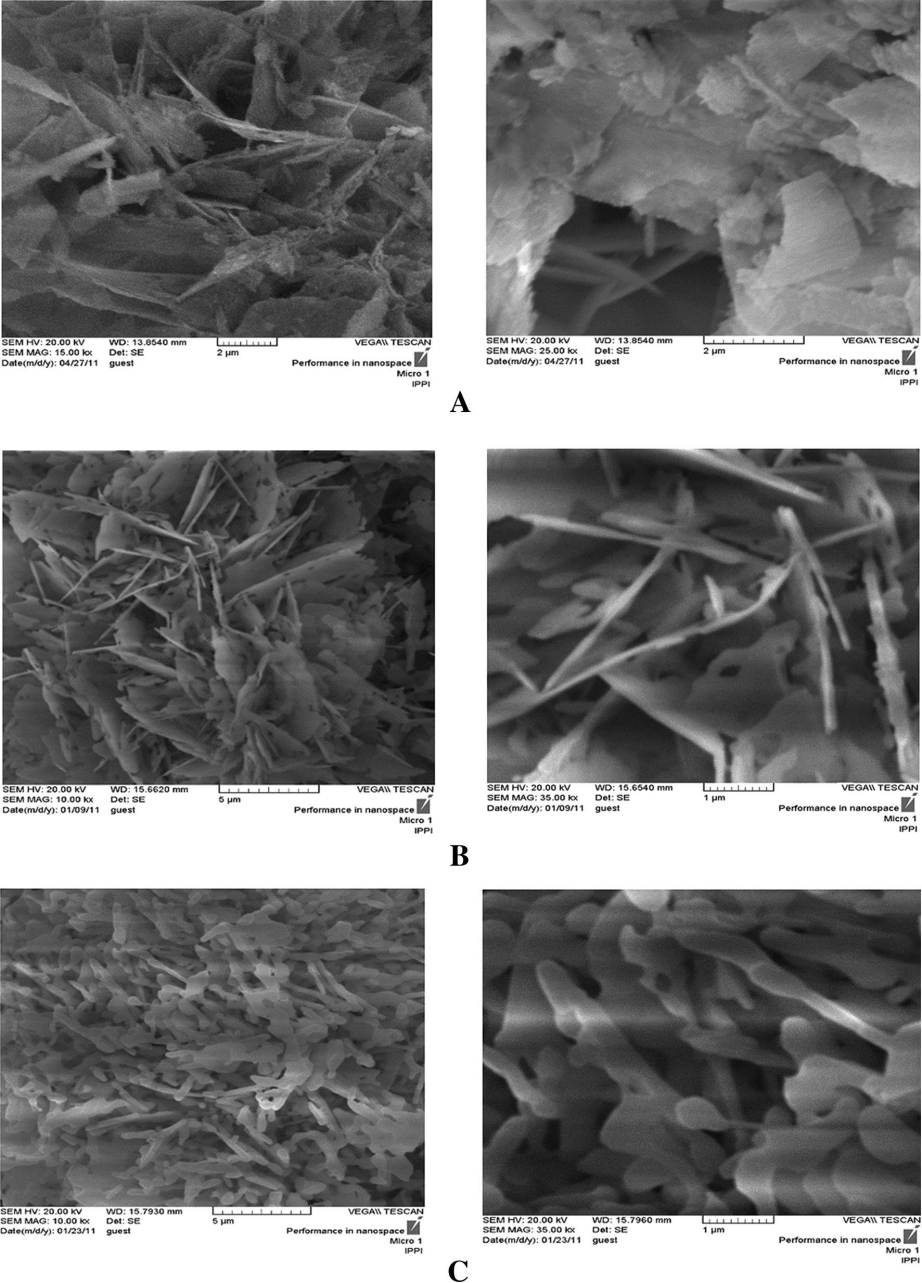
SEM photomicrographs of pre-calcined TCP (**a**), nano-TCP-1 (**b**) and nano-TCP-2 (**c**)

In this study a simple method is utilized for synthesis of nano-sized β-TCP powders with plate-like morphology. These porous, plate-like nano-TCP particles are expected to provide a better interaction with their surrounding environment owing to their larger surface area (Furuzono et al. 2005). Morphology of the as-prepared nano-TCP particles is illustrated in Fig. 1a, showing particles with irregular plate-like morphology. The nano-TCP powder calcined at 800 °C for 5 h (Fig. 1b) was mostly consisted of porous, platelet crystals of 50–100 nm thickness and holes were 100–300 nm in diameter. The calcined crystals were generally smoother in appearance than the pre-calcined plates. The holes in plates of calcined nano-TCP crystal are generated due to a decrease in crystal mass and volume during calcination at 800 °C (Furuzono et al. 2005).

Apatitic tricalcium phosphate, Ca_9_(HPO_4_)(PO_4_)_5_(OH), can be precipitated through the hydrolysis of dicalcium phosphate dihydrate CaHPO_4_·2H_2_O (DCPD) in solution state (Destainville et al. 2003; García Carrodeguas et al. 2003). When Ca/P < 1.5, the initial precipitate mostly consists of DCPD which is slowly hydrolyzed into apatitic tricalcium phosphate. After drying at 80–100 °C, a second phase of anhydrous dicalcium phosphate (DCPA) may also be formed along with the apatitic tricalcium phosphate (Destainville et al. 2003). Apatitic tricalcium phosphate loses a water molecule during calcination and transforms to β-TCP, Ca_3_(PO_4_)_2_, as follows (Destainville et al. 2003):

Ca_9_(HPO_4_)(PO_4_)_5_(OH) → 3Ca_3_(PO_4_)_2_ + H_2_O (> 750 °C).

DCPA can be transformed to calcium pyrophosphate β-CPP, (β-Ca_2_P_2_O_7_), through calcination process according to the following equations:

2CaHPO_4_ → (δ) Ca_2_P_2_O_7_ + H_2_O (450 °C)

(δ) Ca_2_P_2_O_7_ → (β) Ca_2_P_2_O_7_ (850 °C).

The produced β-CPP particle, as impurity, results in the Ca/P lower than 1.5 in the final product.

Comparing SEM micrographs of nano-TCP-1 (Fig. 1b) with nano-TCP-2 (Fig. 1c), it can be concluded that calcination at 800 °C provides particles with lower thickness. Calcination at 900 °C results in particles with higher thickness due to sintering. According to previous studies, sintering of the β-TCP started from 750 °C and its maximum densification occurs between 950 and 1000 °C (Destainville et al. 2003).

**Fig. 2:**
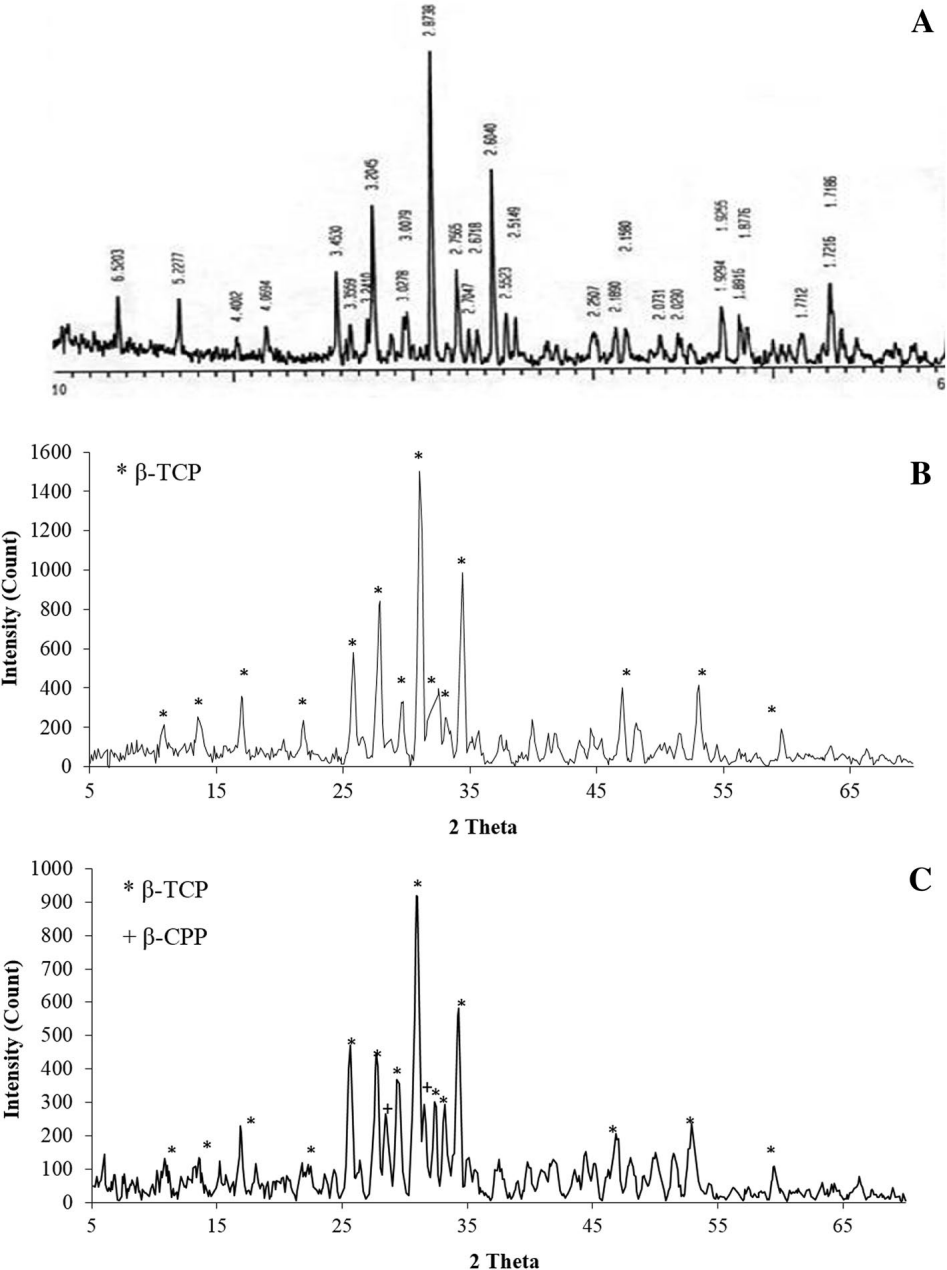
X-ray diffraction pattern of beta-TCP standard card (**a**) (JCPDS No. 09-0169), nano-TCP-1 (**b**) and nano-TCP-2 (**c**)

XRD patterns for the calcined powder, nano-TCP-1, nano-TCP-2, and the TCP standard card are shown in Fig. 2. The diffractograms indicate that the powder mainly comprises of TCP phase. The main characteristic peaks of both nano-TCP-1 and nano-TCP-2 patterns are the same; however, two additional peaks appeared in nano-TCP-2 diffractogram which is attributed to the formation of β-CPP because the Ca/P ratio is lower than 1.5 and the calcination temperature is higher than 850 °C (900 °C) (Destainville et al. 2003). Therefore, calcination at 800 °C is more appropriate for our application as it provides particles with higher porosity and lower thickness.

The Ca/P ratio based on the EDXA results (Table 1) support that the ratio increase from 1.33 to 1.51 with increasing pH from 7.3 (nano-TCP-1) to 8 (nano-TCP-3). For Ca*/*P > 1.50 (e.g. nano-TCP-3), Heughebaert (1977) proposed a thermochemical precipitation mechanism consisting of the following steps which is very close to the apatitic tricalcium phosphate precipitation mechanism. In this way, the first step is the precipitation of an amorphous hydrate compound whose stoichiometry is very close to the TCP:

$$9{\text{Ca}}^{2 + } + 6{\text{PO}}_{4}^{3 - } \to {\text{Ca}}_{9} \left( {{\text{PO}}_{4} } \right)_{6} ,{\text{nH}}_{2} {\text{O}}.$$

This step is followed by the hydrolysis of phosphate ions into hydrogen phosphate and hydroxide ions:

$${\text{PO}}_{4}^{3 - } + {\text{H}}_{2} {\text{O}} \to {\text{HPO}}_{4}^{2 - } + {\text{OH}}^{ - } .$$

The next step corresponds to the appearance of the apatite structure with a Ca/P molar ratio higher than 1.50:

$${\text{Ca}}_{9} \left( {{\text{PO}}_{4} } \right)_{6} + {\text{yCa}}^{2 + } + 2{\text{yOH}}^{ - } \to {\text{Ca}}_{{9 + {\text{y}}}} \left( {{\text{PO}}_{4} } \right)_{6} \left( {\text{OH}} \right)_{{2{\text{y}}}} .$$

For nano-TCP powders with composition ratio of Ca/P > 1.5, hydroxyapatite is formed as a second phase (Chaair et al. 1995; Destainville et al. 2003). In nano-TCP-3 the Ca/P ratio is higher than 1.5; thus it is mainly formed through precipitation of apatitic TCP.

During DCPD crystal growth in aqueous solutions, the dominant face is covered with layers of crystallization water while the lateral faces have a mixed ionic bilayers consisting of phosphate and calcium ions alternate with bilayers of water molecules. Hence, different morphologies of irregular crystals, regular and asymmetric crystals, symmetric crystals, twins, and aggregates are possible to form (Abbona et al. 1993; Sikiric and Füredi-Milhofer 2006). Irregular crystals are asymmetric and mostly occurred in solutions of lower concentration (C ≤ 0.050 M). These crystals are usually flat and thin according to (010), which is their dominant crystal form (Abbona et al. 1993).

For the formation of TCP, DCPD should be transformed into apatitic phosphate through passing a dissolution–precipitation pathway (Tas and Bhaduri 2004). DCPD is the dominant phase at low concentrations due to its low solubility in water (*K*_sp_= 2.04 to 2.39 × 10^−7^) (Kim 2007). Hence, irregular asymmetric crystals and consequently plate-like thin structures are formed (Fig. 1).

To investigate the effect of the ripening time on the ratio of Ca/P, nano-TCP-4 sample was synthesized with 48 h of ripening time. The more prolonged ripening time did not significantly affect the Ca/P ratio (Table 1). It has been reported that the change in Ca/P ratio is mainly observed during the first 8–10 h of ripening (Destainville et al. 2003).

### Demineralization/remineralization studies

**Fig. 3:**
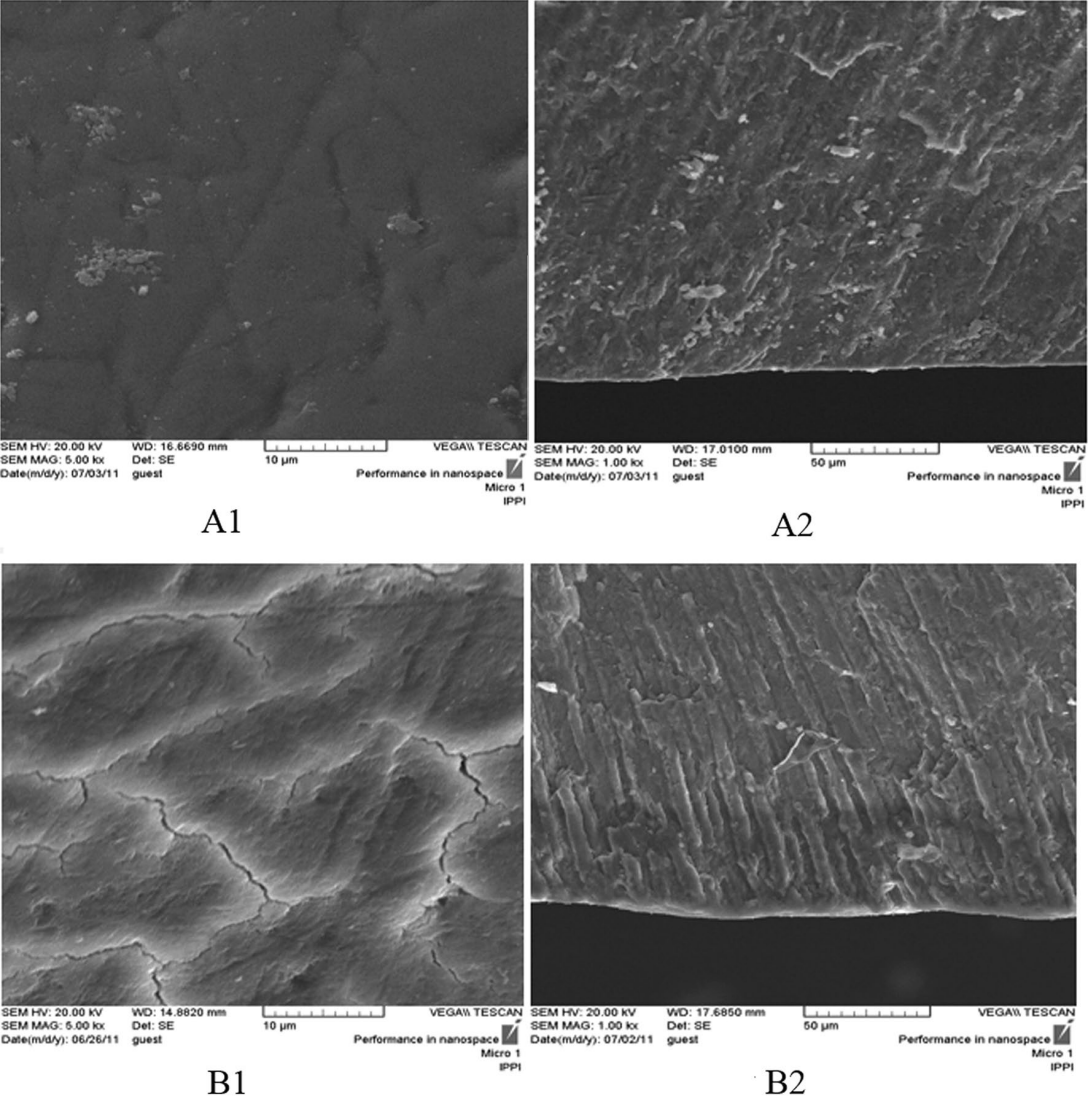
SEM photomicrographs of the natural tooth (**A1**: surface, **A2**: cross-section), and caries induced tooth (**B1**: surface, **B2**: cross-section)

Scanning electron micrographs taken from natural tooth surface and its cross-section are shown in Fig. 3A1 and A2. These micrographs can be compared with the ones obtained after inserting the specimens in demineralizing caries inducing solution (Fig. 3B1 and B2). The surface defects are observed in the form of crack-like lines on the tooth surface after immersion in the demineralization solution. In the cross-sectional view, parallel enamel prisms extending to the surface are observed. The body of the caries lesion can be distinguished from the intact sub-surface enamel, as a darker area. A thin layer on the surface of these specimens separates the sub-surface lesion (Fig. 3B2).

**Fig. 4:**
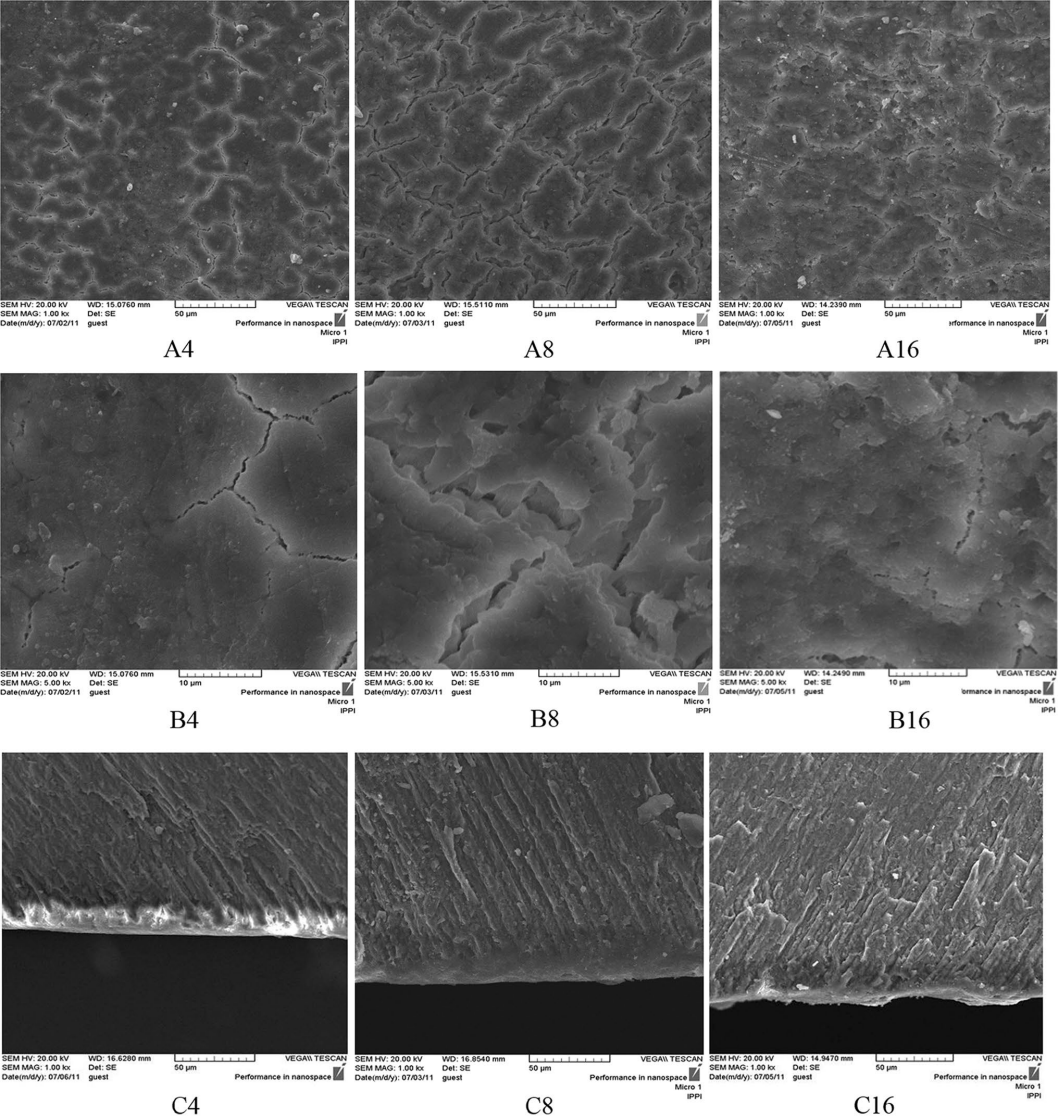
SEM photomicrographs of the teeth treated with NaF 0.05 wt% solution (Group I, positive control). The **a**–**c** codes stand for micrographs obtained from the tooth surface (× 1000 mag.), tooth surface (× 5000 mag.) and tooth cross-section (× 1000 magnification). The digits (4, 8, and 16) after alphabets denote the incubation time in days

In the TCP experimental groups (III, IV, and V) after 4 days pH cycling, the crack lines formed on the surface after demineralizing process have disappeared and are covered by a homogenous layer according to SEM micrographs (Figs. 6, 7, 8A4, B4). In the positive control NaF group (I) on day 4, remarkable changes are not seen on the surface and the crack lines are still observable (Fig. 4A4 and B4). From day 8 of pH cycling, the teeth surface has become globular in appearance in the TCP experimental groups (III, IV, and V) (Figs. 6, 7, 8A8, B8); and positive control NaF group (I) begins to show some changes in the surface morphology (Fig. 4A8 and B8). On day 16 of pH cycling, in all experimental groups (I, III, IV, and V) a globular surface with fine and homogenous appearance is observed, although in group I (NaF) some crack lines are still observed (Figs. 4, 6, 7, 8A16, B16).

**Fig. 5:**
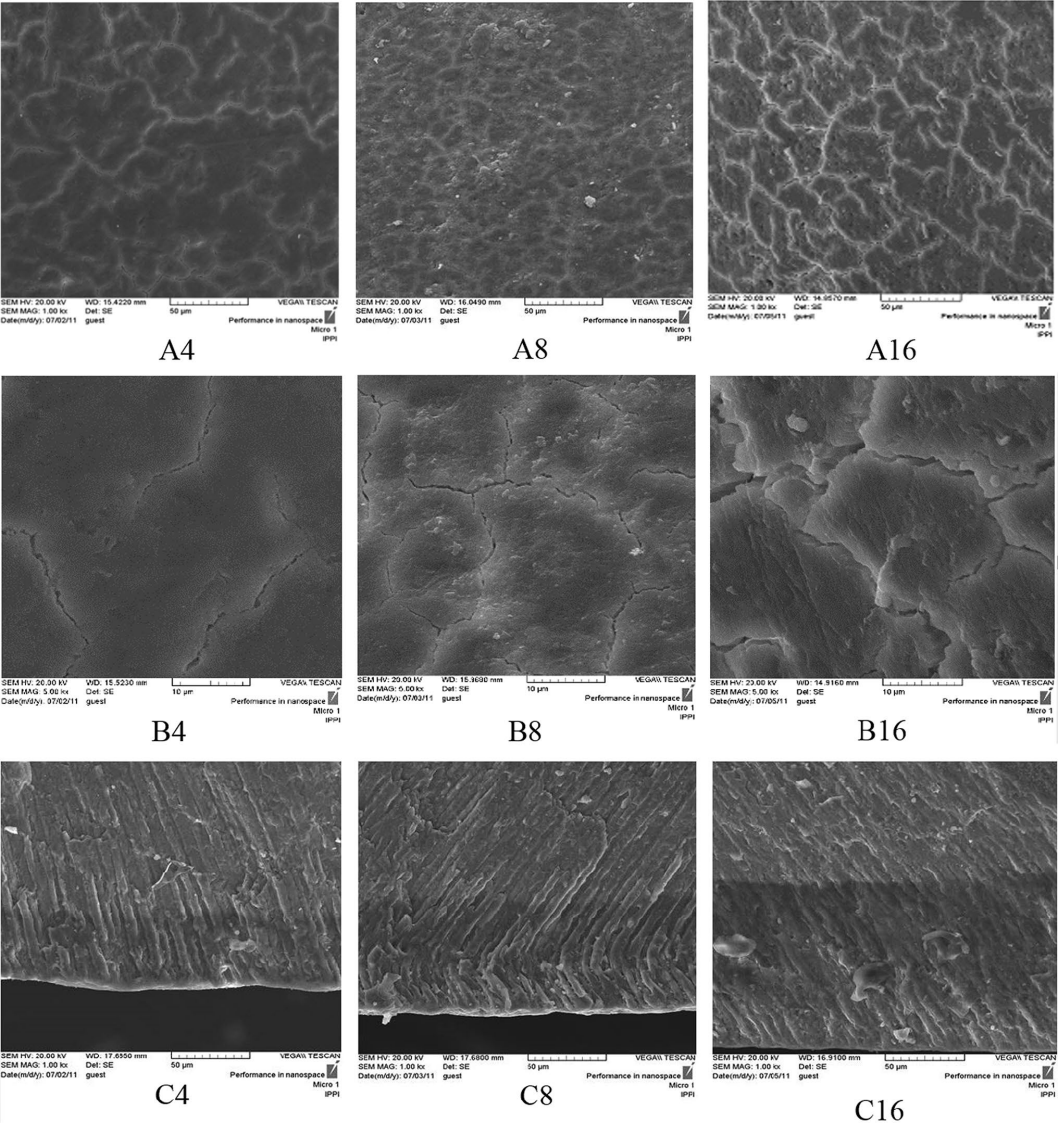
SEM photomicrographs of the teeth treated with nano-TCP 0 wt% solution (Group II, negative control). The **a**–**c** codes stand for micrographs obtained from the tooth surface (× 1000 mag.), tooth surface (× 5000 mag.), and tooth cross-section (× 1000 mag.). The digits (4, 8, and 16) after alphabets denote the incubation time in days

**Fig. 6:**
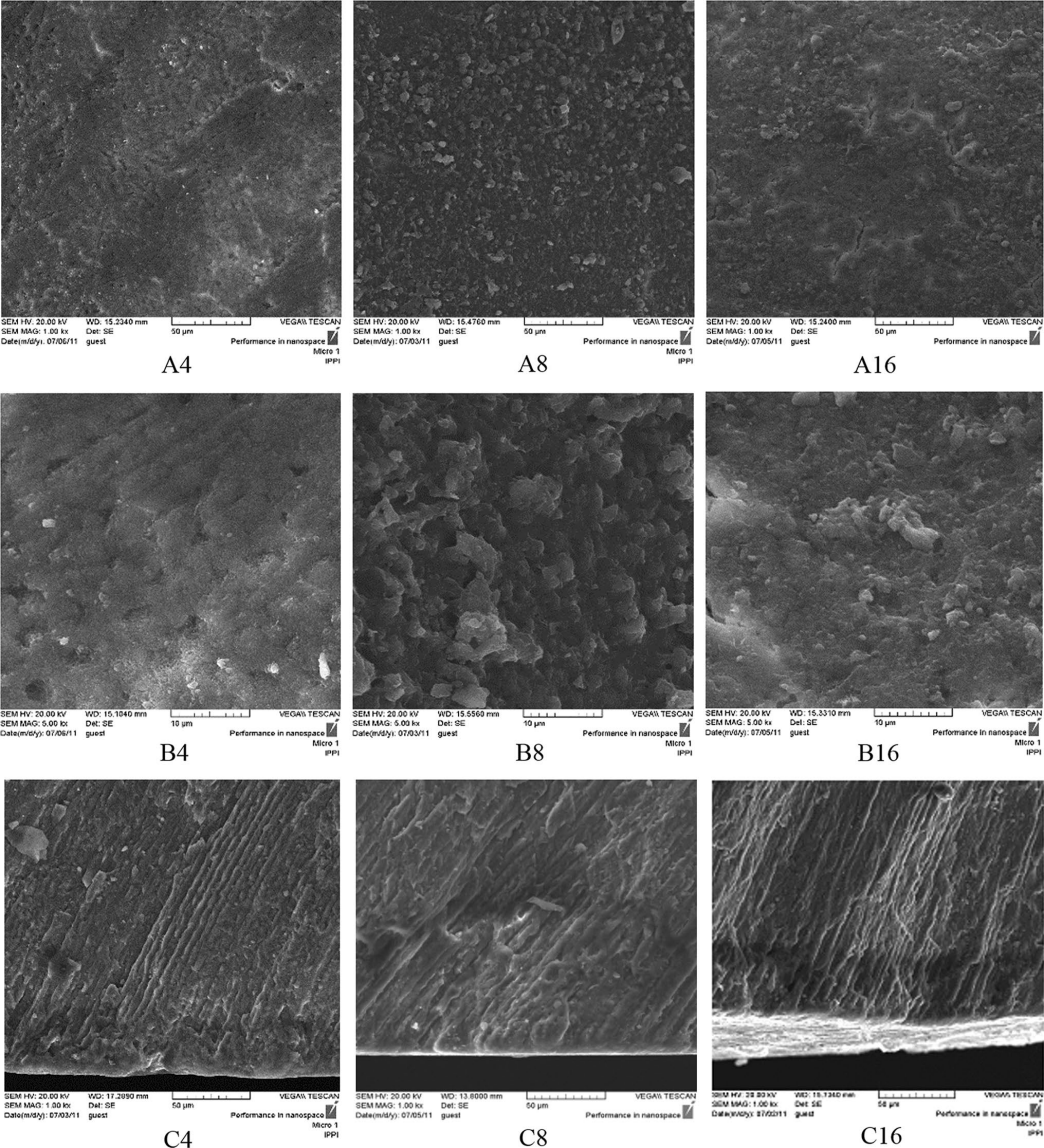
SEM photomicrographs of the teeth treated with nano-TCP 1 wt% solution (Group III). The **a**–**c** codes stand for micrographs obtained from the tooth surface (× 1000 mag.), tooth surface (× 5000 mag.), and tooth cross-section (× 1000 mag.). The digits (4, 8, and 16) after alphabets denote the incubation time in days

**Fig. 7:**
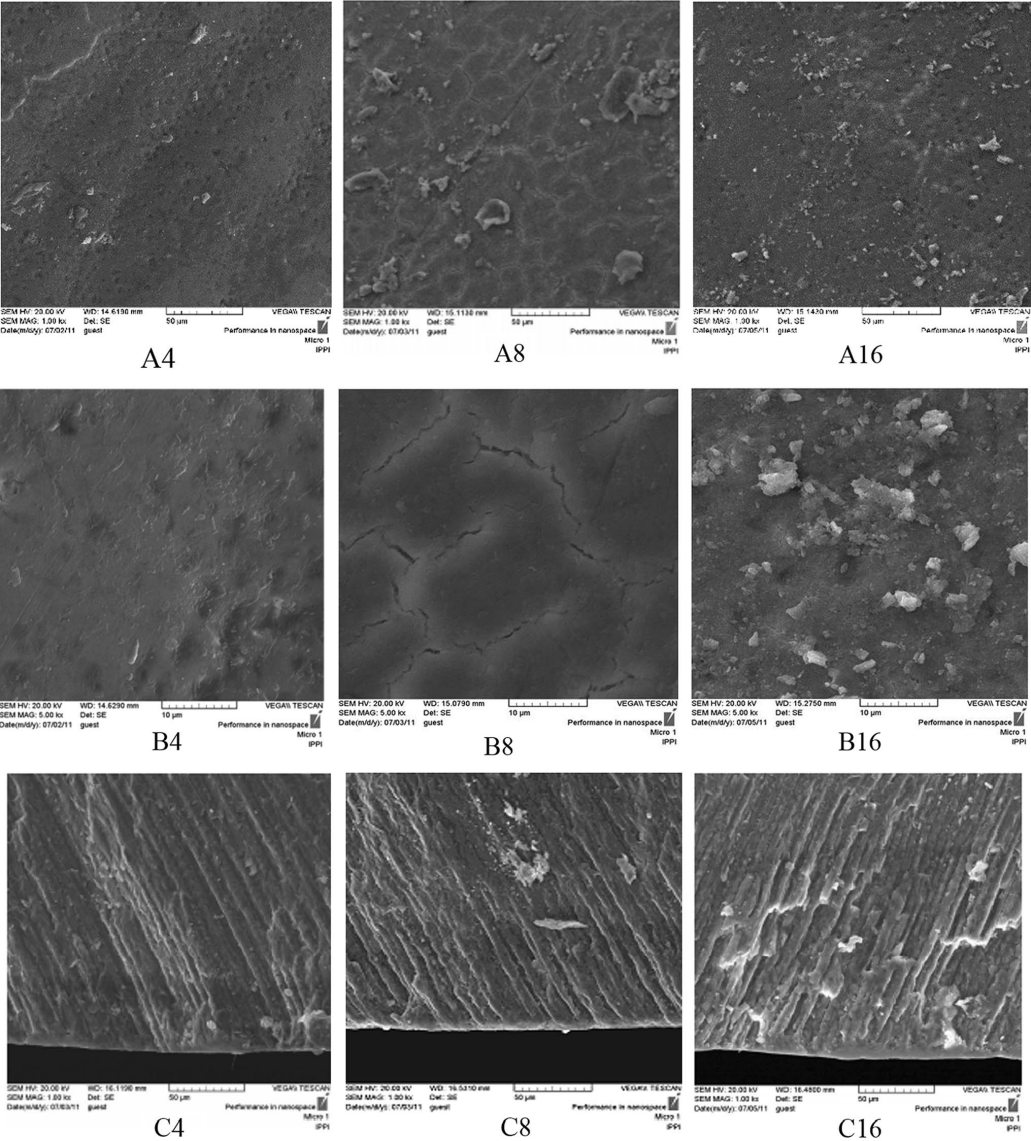
SEM photomicrographs of the teeth treated with nano-TCP 3 wt% (Group IV). The **a**–**c** codes stand for micrographs obtained from the tooth surface (× 1000 mag.), tooth surface (× 5000 mag.), and tooth cross-section (× 1000 mag.). The digits (4, 8, and 16) after alphabets denote the incubation time in days

**Fig. 8:**
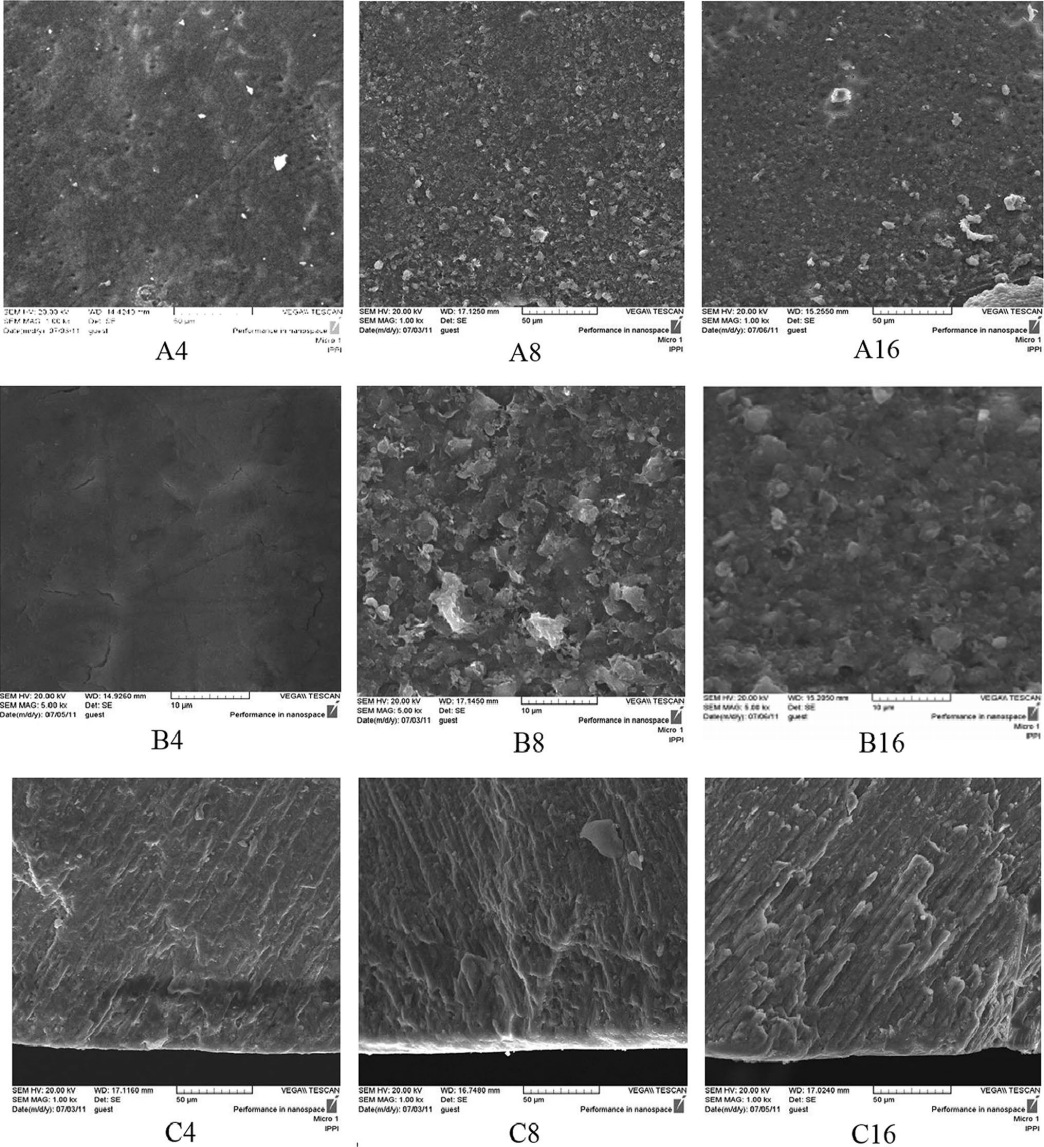
SEM photomicrographs of the teeth treated with nano-TCP 5 wt% (Group V). The **a**–**c** codes stand for micrographs obtained from the tooth surface (× 1000 mag.), tooth surface (× 5000 mag.), and tooth cross-section (× 1000 mag.). The digits (4, 8, and 16) after alphabets denote the incubation time in days

In the cross-section view of the specimens, a thin distinct surface layer was observed on day 4 of pH cycling in the TCP and NaF groups (I, III, IV and V) (Figs. 4, 6, 7, 8C4) which became more distinct after day 8, especially in group I (NaF, positive control) (Figs. 4, 6, 7, 8C8). On day 16 of pH cycling, the thin surface layer is still thicker in group I (Fig. 6C16). In addition, in TCP groups (III, IV and V), the thickness of the surface layer increased with the increase in nano-TCP solution concentration (Figs. 6, 7, 8C16). However, this increase is less than group I (NaF) (Fig. 4C16). The SEM micrographs of the surface of the specimens in group II (negative control) showed that the crack lines became more distinct with time (Fig. 5A, B). Also, in the cross-section view of the caries lesion, the depth has increased with time (Fig. 5C).

Surface layer thicknesses in the experimental groups (Groups I–V) are tabulated in Table 2. Analyzing these findings using two-way ANOVA indicates a statistically significant difference in surface layer thickness between different concentrations of TCP solutions (*F* = 19.82, *p *= 0.00). Besides, the surface layer thickness showed a statistically significant difference (*F* = 68.96, *p *= 0.00) between different time groups.

**Table 2:** Surface layer thickness (mean ± SD, in micrometer) as a function of incubation time and solution concentration observed in I–V test groups

Time (day)	Group I: positive control^a^	Group II: negative control^b^	Group III: 1 wt%/vol% nano-TCP	Group IV: 3 wt%/vol% nano-TCP	Group V: 5 wt%/vol% nano-TCP	Total
4	10.5 ± 1.3 (*n *= 3)	9.0 ± 1.6 (*n *= 3)	4.5 ± 0.8 (*n *= 3)	9.5 ± 1.9 (*n *= 3)	10.0 ± 0.2 (*n *= 3)	8.7 ± 2.5 (*n *= 15)
8	13.9 ± 0.6 (*n *= 3)	10.0 ± 1.2 (*n *= 3)	7.0 ± 1.1 (*n *= 3)	5.5 ± 0.3 (*n *= 3)	8.2 ± 0.5 (*n *= 3)	9.0 ± 3.1 (*n *= 15)
16	6.3 ± 0.2 (*n *= 3)	11.0 ± 1.3 (*n *= 3)	15.5 ± 0.4 (*n *= 3)	8.5 ± 0.5 (*n *= 3)	19.8 ± 2.4 (*n *= 3)	12.2 ± 5.2 (*n *= 15)

^a^0.05 wt% NaF solution

^b^0 wt% nano-TCP solution

Depth of the lesion was also investigated using SEM micrographs and its corresponding values are listed in Table 3 as a function of incubation time and TCP solution concentration. The two-way ANOVA analyses indicated statistically significant difference in caries lesion depth between different TCP solution concentrations (*F* = 1480.40, *p *= 0.00). In addition, a statistically significant difference (*F* = 1699.70, *p *= 0.00) was also found in caries lesion depth between different time groups (4, 8 and 16 days). In negative control group, changes observed in the lesion depth show a steadily increasing trend with time. In other words, the incipient enamel caries lesion depth increases with time from 0 to 16 days. In positive control group; however, the lesion depth does not show considerable change. Increasing the lesion depth with time is remarkable in TCP experimental groups that is less than negative control and more than positive control group.

**Table 3:** Depth of the lesion (mean ± SD, in micrometer) as a function of incubation time and solution concentration observed in I–V test groups

Time (day)	Group I: positive control^a^	Group II: negative control^b^	Group III: 1 wt%/vol% nano-TCP	Group IV: 3 wt%/vol% nano-TCP	Group V: 5 wt%/vol% nano-TCP	Total
4	23.8 ± 0.9 (*n *= 3)	51.4 ± 0.9 (*n *= 3)	45.0 ± 0.9 (*n *= 3)	38.5 ± 2.6 (*n *= 3)	41.3 ± 0.7 (*n *= 3)	40.0 ± 9.6 (*n *= 15)
8	25.8 ± 1.4 (*n *= 3)	79.6 ± 1.2 (*n *= 3)	39.0 ± 0.9 (*n *= 3)	67.6 ± 2.4 (*n *= 3)	64.9 ± 0.5 (*n *= 3)	55.4 ± 20.6 (*n *= 15)
16	25.6 ± 0.2 (*n *= 3)	126.6 ± 2.7 (*n *= 3)	101.9 ± 5.1 (*n *= 3)	135.8 ± 1.4 (*n *= 3)	87.3 ± 2.1 (*n *= 3)	95.4 ± 40.4 (*n *= 15)

^a^0.05 wt% NaF solution

^b^0 wt% nano-TCP solution

The weight percentages of calcium and phosphorous elements were determined, using EDXA, on the surface layer, caries lesion, and intact enamel for all experimental groups after 16 days (Table 4).

**Table 4:** Weight percentages of calcium and phosphorous elements of different groups in surface layer, caries lesion, and intact enamel after treatment for 16 days

Test group	Group I: positive control^a^		Group II: negative control^b^		Group III: 1 wt% nano-TCP		Group IV: 3 wt% nano-TCP		Group V: 5 wt% nano-TCP	
Element (%wt)	Ca	P	Ca	P	Ca	P	Ca	P	Ca	P
Surface layer	32.0	16.2	–	–	26.9	17.5	23.3	9.5	25.1	12.0
Caries lesion	23.4	12.8	17.7	9.9	21.1	11.6	26.1	12.6	23.0	12.0
Intact enamel	24.5	13.9	23.6	12.8	26.8	13.5	29.2	14.4	34.3	15.0

^a^0.05 wt% NaF solution

^b^0 wt% nano-TCP solution

Since the purpose of the present study was to investigate the effects of the TCP solutions as mouth-wash on incipient enamel caries lesions, first the substance was prepared at concentrations of 5, 10, and 15 wt% in small experimental amounts as a pilot to analyze the precipitation rate and the possibility of application as mouth wash. Solution concentrations of more than 10 wt% nano-TCP were very viscous to be used as a mouthwash. Hence, solution concentrations of 1, 3, 5 wt% were selected to investigate. The study was conducted as a pH cycling in vitro model, with daily replacement of demineralizing and remineralizing solutions with compositions similar to saliva but different pH. Therefore, the pH changes in the mouth was simulated daily without byproducts accumulation and possibly deactivating the solution (Ten Cate and Duijsters 1982).

The important consideration in creation of enamel caries lesions is that intensive acid invasion results in crystal dissolution and the surface layer is completely destroyed. Whilst the incipient enamel caries lesion is a partial loss of inorganic materials from subsurface enamel, leaving a carious lesion with a well mineralized 20–50 μm surface layer and a subsurface body with 30–50% mineral loss in depth. It is necessary to use weak organic acids in the demineralizing solution to create such lesion. Featherstone (1996) considered lactic or acetic acid as appropriate choice and believed that acids such as citric and hydrochloric would not diffuse into the subsurface and do not simulate the caries process as the weak organic acids. Also, it has been suggested that the carboxymethyl cellulose gel with its calcium binding activity and the presence of calcium, phosphate, and fluoride ions in the solution phase would help preserve the surface layer (Ten Cate and Marsh 1994). The existence of a small amount of fluoride (0.05 ppm) can simulate the remaining fluoride in the saliva after brushing with fluoride toothpaste or mouthwash, or even the fluoride existing in natural saliva if fluoride water has been drunk (Featherstone 2009). Consequently, it can be concluded that this level of fluoride concentration helps preserve the surface layer, though it does not interfere with the treatment responses of remineralization solutions.

The trend of change in the thickness of the surface layer at different time periods shows that in at 0 wt% concentration (negative control), the surface layer thickness is constant at different time periods. The solution concentrations of 1–5 wt% of nano-TCP show a steadily increasing trend (Table 2). The depth of the caries lesion at different time periods, however, shows that at 0 wt% TCP, the depth of the caries lesion increases with time (Fig. 5C). In 0.05 wt% NaF group, the depth of the caries lesions is constant during time periods of the experiment (Fig. 4C). In addition, the increasing trend in the depth of the caries lesion by time is observed in experimental groups of 1, 3, and 5 wt% of nano-TCP, though this increase is less than group 0% and more than group 0.05% NaF (Table 3). It seems that the diffusion and penetration of nano-TCP particles to the body of the lesion is limited.

As can be seen, in the presence of nano-TCP with higher concentrations, a homogenous layer is formed with time progressing on the surface defects created by caries and the crack lines are disappeared (Figs. 7, 8A16, B16). The homogenous layer formed on the surface seems to have a limited thickness and the effect of nano-TCP is limited to the surface. Huang et al. (2009) has noted that hydroxyapatite nano-particles act as scaffolds for further precipitation and absorption of calcium and phosphate ions from the remineralizing solution to the surface of enamel through penetration to porosities. Through this, they can fill up the empty space between the enamel crystals and lead to crystals growth and their integrity. As the penetration of nano-particles into the subsurface areas was not observed, it may be concluded that the calcium and phosphate ions released from the TCP nano-particles form a calcium phosphate structure on the enamel surface which can be different in chemical structure from the primary TCP nano-particles.

In NaF 0.05 wt% group, although the surface defects are still seen (Fig. 4A, B), a homogenous uniform thick layer is observed in the cross section view (Fig. 4C16) which might be attributed to the fluorohydroxy apatite or calcium fluoride. Fejerskov and Kidd (2009) reported that at low concentrations of fluoride (< 50 ppm) and acidic conditions, fluorohydroxy apatite is formed in enamel. However, at high concentrations (> 100 ppm) and acidic conditions, calcium fluoride is likely to form. Accordingly, in the present study, where which NaF concentration is 0.05 wt% (500 ppm), the formed homogenous layer is more probably CaF_2_ (Fejerskov and Kidd 2009).

In the case of fluoride, the mechanism of remineralization is the formation of a fully mineralized layer on the surface and remaining the caries lesion porous. Precipitating the calcium and phosphate ions into a distinct well-mineralized surface layer, fluoride prevents leaching off the ions released during demineralization of enamel. With the progressing of time and reduction of permeability of this layer, the penetration of further ions through the formed layer is decreased leaving a porous sub-surface area (Fejerskov and Kidd 2009; White 1987).

Analyzing the SEM micrographs of nano-TCP specimens (Figs. 6, 7, 8), it is concluded that an impermeable layer is rapidly formed on the enamel surface which covers the surface defects and limits the access of calcium and phosphate ions to the subsurface area. In the case of NaF (Fig. 4), however, the impermeable layer is formed slowly which provides more time for the ions to penetrate and access to the subsurface. It has also been reported that the layer formed by NaF is still permeable towards the Ca–P ions which let the ions diffuse to the deeper areas of caries lesion (Nishio et al. 2006).

The depth of demineralization has increased in the 0 wt% solution with time (Fig. 5C). Since this sample is exposed to H^+^ ions of demineralizing solution in the pH cycling regime, it is obvious that the ion penetration continued during the experiment increasing the depth of caries within 16 days. Despite the existence of the H^+^ ions in NaF group, there is no increase in the depth of the caries (Fig. 4C). Although NaF makes a physical barrier on the surface, it is not well impermeable towards the H^+^ ions. The prevention property of the NaF might be attributed to the chemical capability of fluoride in precipitation of Ca–P ions released during demineralization on enamel crystals, preventing ions leaching out the tooth. In the nano-TCP groups, preventing the increase in caries depth is not as much as the NaF group (Table 3). These solutions have shown a reduction in the mineral loss rate in comparison with 0 wt% group. However, this reduction is less than NaF group. Consequently, NaF has a better capacity in restraining demineralization.

Huang (Huang et al. 2009) reported that the solubility of hydroxyapatite is not enough to provide sufficient Ca–P ions to prevent enamel demineralization. TCP, however, is more soluble and, consequently, may provide more ions to suppress the demineralization of the enamel, as seen in the specimens treated with the TCP solutions (Figs. 6, 7, 8C, Table 4).

Karlinsey et al. (2009a) reported that the calcium-phosphate compounds as bio-available and remineralizing components, incorporated into some fluoride-containing dental products, interact synergistically with the fluoride. The problem of mixing these components in a single product is that TCP produces CaF_2_ in the presence of fluoride and is not able to provide the necessary calcium and phosphate ions for the enamel remineralization (Karlinsey and Pfarrer 2012; Karlinsey et al. 2009a, b). Although most of the studies have shown surface and subsurface re-hardening of caries lesions, a trend for a homogenous mineral matrix formation is essentially seen in approximately 75 μm from the enamel surface (Karlinsey et al. 2011a) that differs from our observations (Table 2). It has been reported that enamel remineralization is the same for silica-based dentifrices with 1100 ppm F and 500 ppm F plus functionalized TCP up to 37.5 μm at which the remineralization trend shows improvement in TCP groups up to about 70 μm (Karlinsey et al. 2011b; Karlinsey and Mackey 2009).

In our findings, although the nano-particles may improve penetration and diffusion into the subsurface, it seems that further penetration of nano TCP particles is inhibited due to the formation of a stabilized condensed outer layer of calcium phosphate compounds that limits the remineralization to the surface. The precipitation of the calcium-phosphate ions on surface may be more efficient in porous enamel than in remineralization of incipient enamel caries lesion. The resistance of enamel increases by closing these surface porosities. This hypothesis is supported by the study conducted on the effect of TCP on the reduction of the reversible color changes after bleaching (Brown et al. 1977). According to the mechanism of occluding dentin tubules, TCP would be a promising material in treatment of dentin hypersensitivity (Karlinsey et al. 2011a).

## Conclusions

Porous plate-like nano-TCP crystals of 50–100 nm thickness were synthesized though a wet chemical method and calcination at 800 °C. Because of nano-sized thickness of the particles with plate-like morphology, they are sintered easily at 900 °C resulting in higher particle thickness. The content of the HAp and β-CCP in the nano-TCP, size and morphology of the particles could be controlled mainly by precursor concentration, second-phase addition rate, and solution pH. The prepared nano-sized β-TCP particles with plate-like morphology are more appropriate for dental remineralization due to the larger surface area to release ions.

In the case of stimulating remineralization among different nano-TCP concentrations, 1 and 3 wt% solutions showed a significant difference. The remineralization effect of the solutions increased with time. The NaF 0.05 wt% solution proved to be more effective than other solutions in preventing demineralization.
